# Statistical characteristics of comic panel viewing times

**DOI:** 10.1038/s41598-023-47120-w

**Published:** 2023-11-20

**Authors:** Hikaru Ikuta, Leslie Wöhler, Kiyoharu Aizawa

**Affiliations:** 1 Department of Information and Communication Engineering, The University of Tokyo, Tokyo, 113-8656 Japan; 2 The University of Tokyo JSPS International Research Fellow, Tokyo, 113-8656 Japan

**Keywords:** Computer science, Human behaviour

## Abstract

Comics are a bimodal form of art involving a mixture of text and images. Since comics require a combination of various cognitive processes to comprehend their contents, the analysis of human comic reading behavior sheds light on how humans process such bimodal forms of media. In this paper, we particularly focus on the viewing times of each comic panel as a quantitative measure of attention, and analyze the statistical characteristics of the distributions of comic panel viewing times. We create a user interface that presents comics in a panel-wise manner, and measure the viewing times of each panel through a user study experiment. We collected data from 18 participants reading 7 comic book volumes resulting in over 99,000 viewing time data points, which will be released publicly. The results show that the average viewing times are proportional to the text length contained in the panel’s speech bubbles, with a rate of proportion differing for each reader, despite the bimodal setting. Additionally, we find that the viewing time for all users follows a common heavy-tailed distribution.

## Introduction

Written language and visual stimuli serve a large purpose in everyday human life. Combining both of these modes of information, comics are a widespread form of media used for various purposes including art and advertisement. Due to the bimodal nature of comics, we expect that the analysis of human comic reading behavior shall provide insights into how humans simultaneously process various kinds of stimuli.

In this paper, we particularly focus on the viewing times of each comic panel as a measurable indicator of the information contained in the panel, and investigate the statistical properties of the comic panel viewing times. The panel viewing times are measured using a panel-wise comic viewer designed for our experiment. We have conducted a user study and obtained a dataset containing over 99,000 raw panel viewing time data points, which we will be releasing publicly.

Recently, due to the popularity of viewing comics on handheld devices, a new style of comics with a vertical layout is gaining widespread popularity^1^. A notable characteristic of vertical comics compared to conventional comics is that the layout is linear. While conventional comics potentially allow the reader to skip panels through free viewing, the layout of vertical comics is expected to let the user only transition between neighboring panels at a time. Therefore, our results not only provide insights regarding the processing of bimodal information but could also inform the future design of comic readers, e.g., by adding automatic panel transitions based on the viewing time.

Our contributions and findings are the following: (1) we find that even in a bimodal setting where both images and text are concurrently present, the average comic panel viewing times are proportional to the length of the text contained in the panel, (2) we find that the distribution of comic panel viewing times share a similar heavy-tailed form between all readers, and (3) we will be releasing the comic panel viewing time dataset collected from our user study experiment.

## Related work

The human processing of bimodal information consisting of text and images has been studied in contexts including infographics^2^, instruction manuals^3^, advertising^4^, and online articles^5^. In this work, we set out to investigate the reading behavior of participants reading whole volumes of comic books and analyze the influence of image and text on the viewing time.

### Panel-wise comic viewing interface

The concept of a panel-wise comic viewer has been proposed in the past in various contexts. Yamada et al.^6^ propose an interface for classical cell phones, where the display size and the bandwidth are heavily limited. In their work, since the image becomes resized too small to the point where the text becomes unreadable, the text is shown in a different region along with the panel image.

The demand for reading comics on handheld devices still continues today in the context of modern hardware. Due to the high prevalence of smartphones, it is getting increasingly common for comics to assume the limited display widths of smartphones beforehand at the time of content creation^1^. Such comics are drawn so that panel orders advance vertically for most panels, and panels are rarely arranged horizontally. This smartphone-oriented panel structure usually demands the user to scroll the screen every time the user wishes to view the next panel.

**Figure 1:**
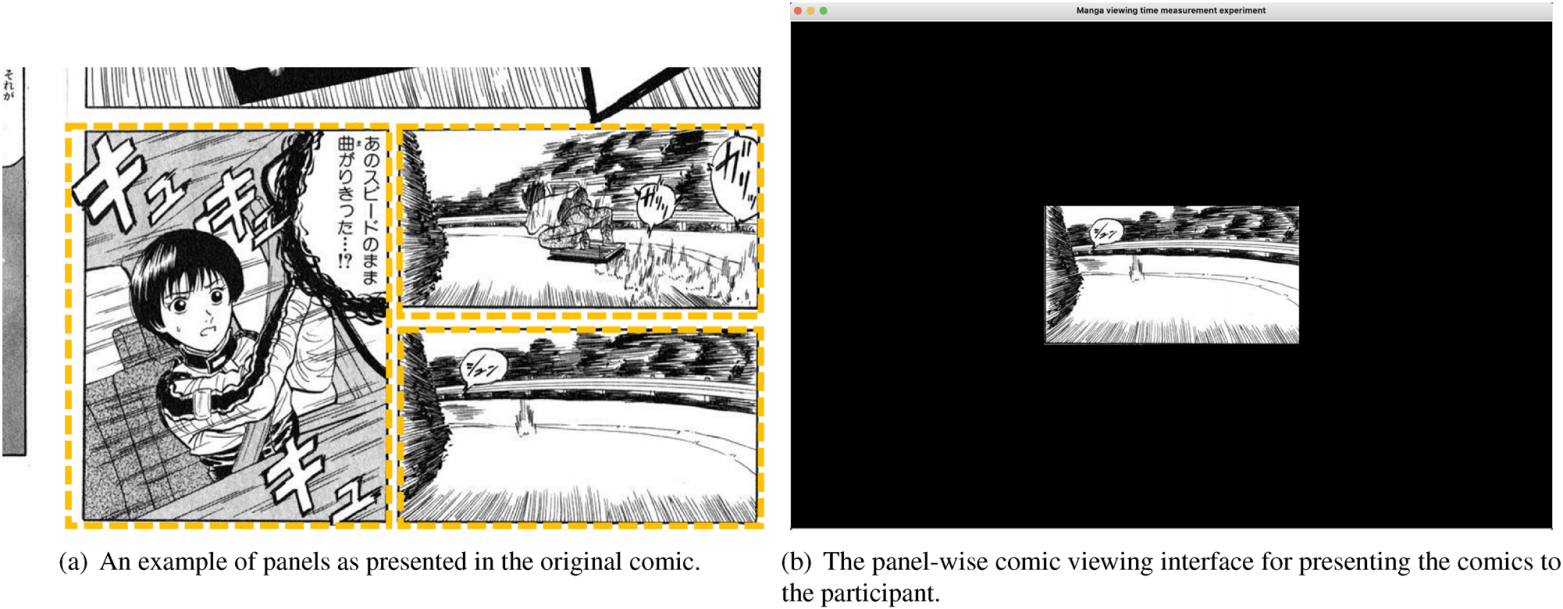
(**a**) Panels are cropped for being shown in the user interface. The dotted lines indicate the region presented to the reader in the panel-wise comic viewing interface used in the user study experiment. (**b**) Panels are navigated using the arrow keys on the keyboard. The comic panels in the figure are referenced from the comic *Saisoku!! Vol. 1* by Naomasa Matsuda, included as a part of the Manga109 dataset^17,18^. The comic panels are used under the permission of the author Naomasa Matsuda. Please see https://www.manga109.org/ for more details.

In order to record the viewing times of each panel, we create a comic viewing interface where each panel is shown individually, as shown in Fig. 1. Therefore, our panel-wise viewing interface closely resembles vertically oriented comics drawn for smartphones, allowing insights into their usage and possible future functionalities such as automatic transitions instead of manual scrolling.

### Comic panel viewing times

Cohn^7^ investigates the viewing times of comic panels for 12 participants. The stimulus used for the experiment were 180 black and white comic strips which all had a four-panel structure with consistent panel sizes. Among the four experiments performed in their work, one experiment focuses on the impact of partially reordering four-panel comics, where the viewing time of these four-panel comic strips were measured. Notably, the comics used in their work mostly consist of no text which does not reflect the bimodal nature of most comics. Similarly, Cohn and Wittenberg^8^ investigate the effect of special symbolic comic panels called *action stars*, where they measure and compare the viewing times of short comic strips with various modifications related to action star panels. Magliano et al.^9^ measure the viewing time of four-panel comic strips, for the purpose of evaluating how long it took participants to infer undepicted events that happen between the comic panels.

In contrast to these works, we investigate the bimodal aspects of comic reading by analyzing the viewing times of participants in relation to the amount of text shown in panels. Furthermore, our stimuli consists of whole comic volumes instead of short comic strips allowing us to create a more natural reading experience for participants and to gather more data.

### Gaze measurement

Many works focus on gaze measurement of comic books and other forms of artwork. Foulsham et al.^10^ use 6-panel comic strips and measure the gaze sequence when viewed by human participants. Rohan et al.^11^ use eye tracking in comics with various modifications of the depiction of onomatopoeia, to compare the effectiveness of various methods of translating Japanese comic onomatopoeia to English. Eakta et al.^12^ measure the gaze position and durations of humans when viewing photos and paintings, where their work mainly focuses on spatial features such as salient regions. Hutson et al,^13^ use an eye tracker to measure the gaze sequence of 6-panel comic strips to investigate the regions of interest, number of fixations, and the fixation durations during comic comprehension. Laubrock and Dunst^14^ summarize various works related to computational approaches to comics.

Several works combine eye tracker data and generative models for content creation. Thirunarayanan et al.^15^ use eye tracker data to automatically create dynamic comic effects. Cao et al.^16^ use eye tracker data to create layouts.

While eye tracking studies enable insights into reading behavior, they generally need to be done in-situ as specialized hardware and supervision are required to obtain accurate eye tracking data. In contrast to this, we collect large amounts of data from a natural reading environment which can give a better representation of the usual comic reading behavior of our participants.

## Materials and methods

In order to investigate comic reading behavior, we invite participants to an experiment in which they read whole comic book volumes in a panel-wise reader while we are recording their viewing times for each panel.

### Apparatus

To measure the viewing time of each panel, the comic viewing interface made for this experiment is designed to show the comics in a panel-wise manner. Before the experiment, each comic panel is cropped as shown in Fig. 1a. These panels are shown in a panel-wise comic viewing interface shown in Fig. 1b. The user can make transitions between neighboring panels using the arrow keys on the keyboard.

The interface has two phases, the title selection phase and the comic viewing phase. The interface first starts with the title selection phase, where the program prompts the user to select a comic to read. After selecting a title, the interface transitions into the comic panel viewing phase where the user navigates through the comic.

At the beginning of the comic panel viewing phase, the interface first presents an intermediate screen for the user to prepare for viewing the comic. When the user presses either the left or right arrow key on the keyboard, the experiment begins and the actual comic panels from the data are shown. During the viewing phase, the user has three operations available: navigate to the next panel, navigate to the previous panel, and suspend the browsing phase. Navigating to the next and previous panels is done by using the arrow keys on the keyboard. Suspending the browsing phase was done by either pressing the Escape key on the keyboard or by closing the window with the mouse. After suspending, the participant is able to resume the viewing phase at any time. Every time the user executes an operation, the comic panel viewer records the timestamp of each event measured in milliseconds.

In order to preserve the original comic reading experience to its maximum extent, the size of the panels is adjusted in a way that attempts to retain both the relative proportion and the content readability as much as possible. For each comic volume, the resizing procedure uses the same resizing ratio for all panels, unless panel sizes would exceed the limits of the screen in which case they are reduced. As a result, 96% of the panels are resized using the same resizing rate and only 4% of the panels are resized disproportionally from the original work.

The resizing procedure can briefly be described as follows. We first define the base resizing ratio $r_{\textrm{base}}$ as

1 $$\begin{aligned} r_{\textrm{base}} = \min \left( \frac{w_{\textrm{base}}}{\overline{W}}, \frac{h_{\textrm{base}}}{\overline{H}} \right) . \end{aligned}$$

where $\overline{W}$ and $\overline{H}$ are the mean panel width and height for the given comic, $w_{\textrm{base}}$ and $h_{\textrm{base}}$ are the base panel width and height defined as 600 px and 400 px, respectively.

Next, each panel is attempted to be resized with the ratio $r_{\textrm{base}}$. When doing so, we check if either the width or the height exceeds the main viewport width and height *W* and *H*, defined as 1180 px and 780 px, respectively. *W* and *H* show the size of the black region shown in Fig. 1b. When the panel exceeds the main viewport size, the panel is resized so that the longest dimension fits within the viewport size (*W*, *H*). This allows the relative panel sizes to be preserved as much as possible from the original work.

### Materials

All comic images used in this experiment are obtained from the Manga109 dataset^17,18^. 7 comics shown in Table 1 were selected from the Manga109 dataset for the experiment. The titles here were selected to represent a broad range of genres and panel layouts. For example, we have included the Four-frame Cartoons genre, which has a highly regular layout. In this genre, most of the panels are of the same size and are arranged in the same layout. All comics are written in Japanese. The total number of panels shown to each participant in the experiment was 5,539 panels. The number of panels for each title is shown in Table 1. Additionally, we used the first 33 panels from *Utyukakatsuki Eva Lady, Vol. 1* written by Shii Miyone to illustrate the usage of the interface.

**Table 1:** Statistics of the comics used in the panel-wise comic viewing experiment.

Title	Author	Genre	Panels
Saisoku!! Vol. 1	Naomasa Matsuda	Sports	924
ARMS	Masaki Kato	Science Fiction	615
Dual Justice Vol. 1	Yusuke Takeyama	Battle	802
Everyday Osakana-chan	Yuka Kuniki	Animals	941
Nichijou Soup	Uni Shindou	Comedy	916
Unbalance Tokyo	Minako Uchida	Science Fiction	971
Youchien Boueigumi	Tenya	Comedy, Four-frame Cartoons	370
Total			5,539

The panel bounding boxes are based on the annotations from the Manga109 dataset, with manual edits for the experiment. In the Manga109 dataset, the panel bounding boxes are defined to be set along the lines of the panel, where objects that stick out of the border of the panels are ignored. For the interface to show such sticking-out objects, we manually re-annotated panel bounding boxes where objects are sticking out.

Since Manga109 does not contain the ground truth panel reading orders, the order of the panels was also annotated as well. The panel order was first determined automatically based on heuristics used in the work by Kovanen et al.^19^, a method used to determine the order of speech bubbles in comics. After the automatic annotation, all of the panel orders were manually inspected and fixed by hand.

### Procedure

The experiment procedure consists of the practice session and the data collection session. During the practice session, we explained the interactions with the framework and ensured that comics were displayed correctly. Afterwards, the participant read through a small amount of sample panels to ensure they understood the usage of the system. The practice session was performed one-on-one or at most two-on-one remotely in an online video session. The data collection session was performed on each participant’s computer remotely without supervision, in order to facilitate a natural comic viewing setting for the participant.

After the practice session, the participant was asked to use the interface to browse the comics selected for the experiment. On every event such as browsing forward or backward, or suspending the experiment to take a break, the user interface records the timestamp of these events. All of the timestamps are recorded in milliseconds. After the participant finishes the experiment, we obtain a time series indicating which panel indices were shown at which time.

After finishing the entire experiment, we additionally asked participants to rate how interesting they found each comic and how easy it was for them to understand on a five-point Likert scale. For each comic, there were questions that asked (a) how interesting the comic was, (b) how comfortable the reading experience on the interface was compared to paper comics, and (c) how understandable the story was when read on the panel-wise interface.

### Participants

After excluding a participant that has read the comics used in the experiment prior to the study, we analyzed the comic panel viewing time data for 18 participants. None of the remaining participants had familiarity with the comics used in the study. Participants were invited using internal communication channels of the university. Since all of the comics used in the experiment were written in Japanese, we required every participant to be fluent in Japanese. Participants received a gift card with a value of 4000 Yen as compensation.

Out of the 18 participants, 17 participants had demographic data available. All of these participants were either bachelor’s or master’s students from The University of Tokyo, where 15 participants were male and 2 participants were female, and spoke Japanese as their first language. The mean age of these participants was 22.12 with a standard deviation of 1.36. The participant without the demographic data is marked with index 17 in the later plots.

The experiment was approved by the Research Ethics Committee of The University of Tokyo, under the approval ID UT-IST-RE-211202-1 and UT-IST-RE-231010. The experiment was performed in accordance with the regulations of the committee for all participants, including the completion of an informed consent form before participating in the experiment.

### Data representation

After collecting the raw viewing timestamp series, the timestamps are converted to the viewing times of each panel. The viewing time of a panel is calculated by taking the difference between neighboring timestamps. An additional case that must be considered is when a participant rewinds panels and views the same panel multiple times. For this case, we simply take the sum of all of the viewing time instances and use that value as the final viewing time. The same holds when the participant suspends the experiment and resumes, when a panel is shown twice.

## Analysis and results

Due to the bimodal nature of comics, it is desirable to decouple the influences of the images and the text during the data analysis. To this end, we perform two analyses that control for the text length.

The first analysis focuses on the relation between the viewing times and the panel’s text length. The text length is defined using the text contained in the speech bubbles, which are available as annotations in the Manga109 dataset. Since all of the texts contained in the dataset are in Japanese, we measure the length of the text as the total number of characters.

The second analysis focuses on the effects of the panels’ image components. For this purpose, we select panels that consist primarily of images instead of text. For each panel, we calculate the sum of the number of characters in all of the speech bubbles within the panel, which we define as the panel’s total text length. We then filtered out panels with a total text length of 0 to 10 and defined them as short-text panels.

### Data overview

**Figure 2:**
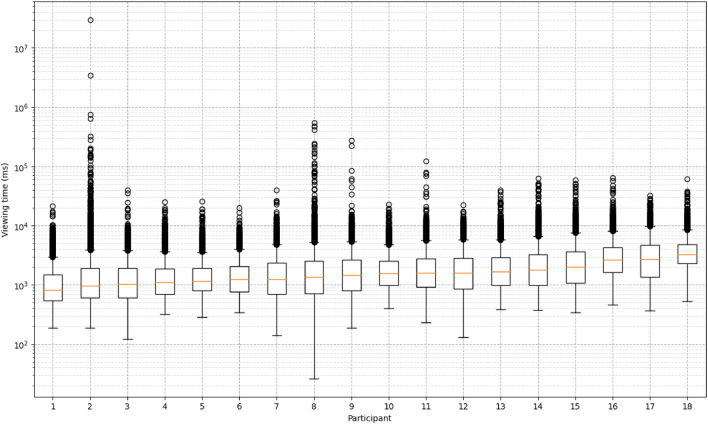
A box plot showing an overview of the entire dataset.

**Table 2:** Average score of the questionnaire over all participants..

Title	(a) Interest to story	(b) Interface usability	(c) Story understandability
Saisoku!! Vol. 1	$3.94 \pm 1.06$	$3.72 \pm 1.18$	$4.28 \pm 0.89$
ARMS	$1.83 \pm 1.20$	$1.78 \pm 1.06$	$1.83 \pm 1.04$
Dual Justice Vol. 1	$4.00 \pm 0.91$	$3.39 \pm 1.14$	$4.28 \pm 0.83$
Everyday Osakana-chan	$3.61 \pm 0.92$	$3.50 \pm 1.20$	$4.00 \pm 1.08$
Nichijou Soup	$2.78 \pm 1.17$	$2.67 \pm 1.14$	$3.39 \pm 1.33$
Unbalance Tokyo	$3.39 \pm 1.29$	$3.11 \pm 1.23$	$3.06 \pm 1.35$
Youchien Boueigumi	$3.11 \pm 1.18$	$3.56 \pm 1.38$	$4.28 \pm 0.96$
Average	$3.24 \pm 1.29$	$3.10 \pm 1.32$	$3.59 \pm 1.35$

The ± sign shows the standard deviation.

Figure 2 shows a box plot showing an overview of the entire dataset. The two largest data points for Participant 2 are over $10^6$ ms, which is over 16 minutes. However, since these panels were ordinary panels with below 55 characters, these two data points were excluded from the later analyses as outliers. Table 2 shows the average score of the questionnaire over all participants.

### Text length dependency

**Figure 3:**
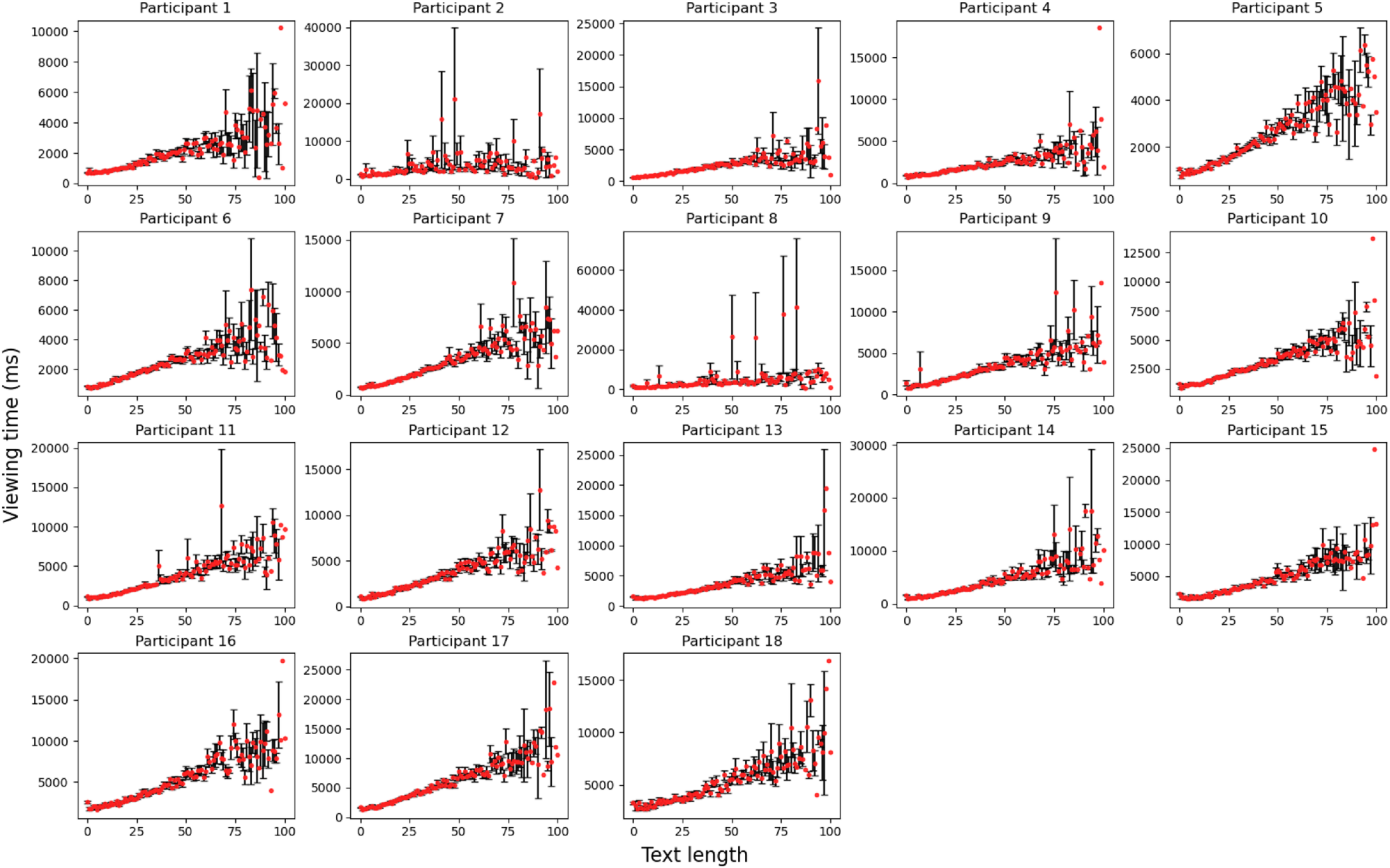
The relation between the panel’s speech bubble text length and the mean viewing times. For each data point, the mean is taken over the panels having the same text length. The error bars show the standard error. The mean viewing time has a strong correlation with the text length.

**Figure 4:**
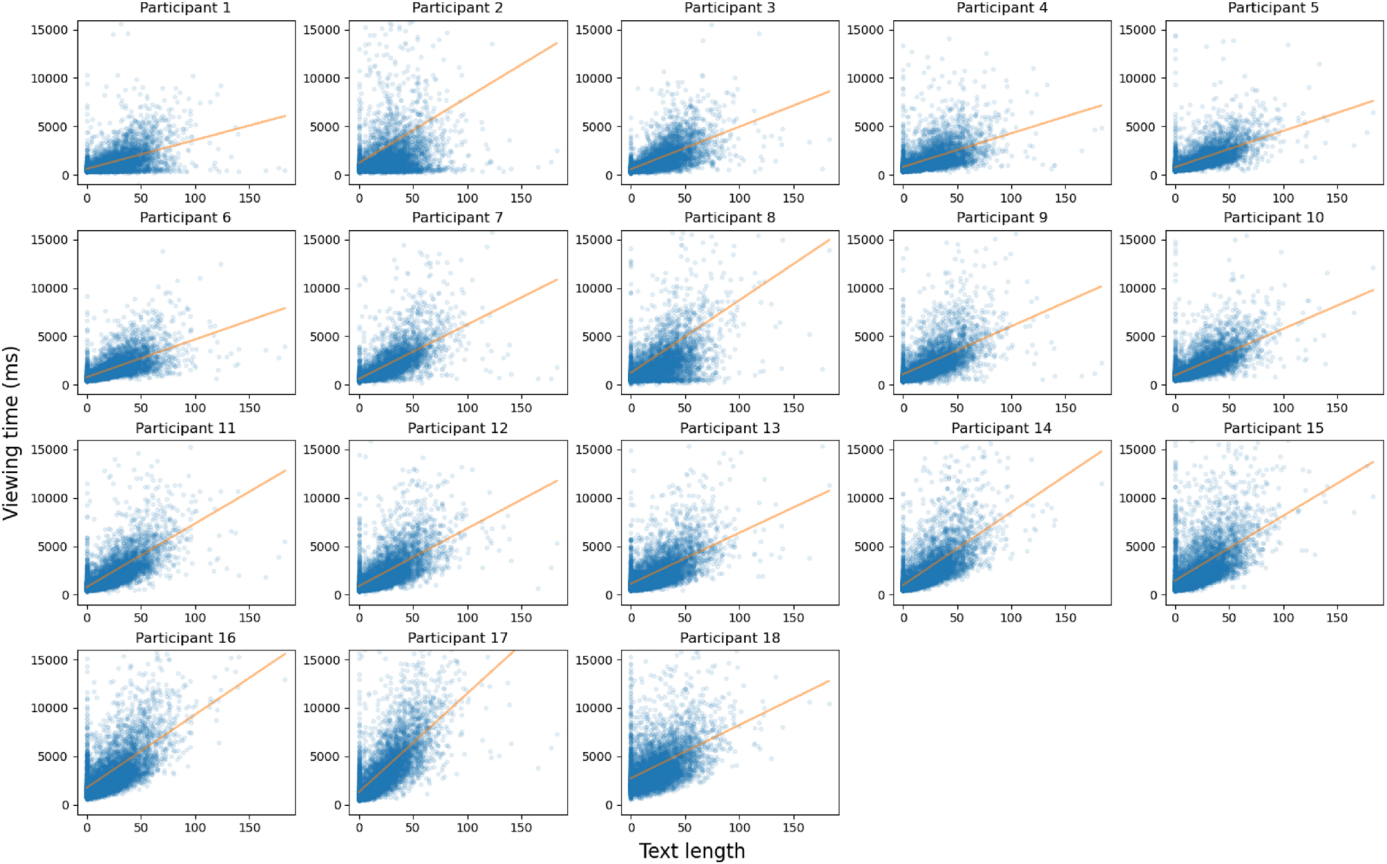
A scatter plot of the panel’s speech bubble text length and the entire viewing time data. The dots show the reading time of each panel, and the line shows the linear regression result.

**Table 3:** Correlations between the text length and panel viewing times.

Participant	Pearson’s R	*p*-value	Spearman’s R	*p*-value
1	0.4805	0.000e+00	0.5424	0.000e+00
2	0.08432	3.291e−10	0.4235	7.731e−240
3	0.5585	0.000e+00	0.7414	0.000e+00
4	0.4687	1.487e−300	0.6489	0.000e+00
5	0.5568	0.000e+00	0.7162	0.000e+00
6	0.6169	0.000e+00	0.7525	0.000e+00
7	0.6235	0.000e+00	0.7875	0.000e+00
8	0.1013	4.14e−14	0.5525	0.000e+00
9	0.1918	4.993e−47	0.7288	0.000e+00
10	0.6098	0.000e+00	0.7214	0.000e+00
11	0.4322	5.408e−251	0.7554	0.000e+00
12	0.6340	0.000e+00	0.7264	0.000e+00
13	0.4986	0.000e+00	0.6352	0.000e+00
14	0.4836	0.000e+00	0.7298	0.000e+00
15	0.4263	1.994e−243	0.6134	0.000e+00
16	0.5145	0.000e+00	0.6200	0.000e+00
17	0.6888	0.000e+00	0.7529	0.000e+00
18	0.4096	4.034e-223	0.4782	0.000e+00
Median	0.4911	−	0.7188	−

Figure 3 shows the relation between the panel’s speech bubble text length and the mean viewing times. Each plot corresponds to each participant. For each data point, the mean is taken over the panels having the same text length. The error bars show the standard error.

Figure 4 shows the entire data along with the linear regression result. Table 3 shows the Pearson and Spearman correlation coefficients between the text length and panel viewing times. The median Pearson correlation coefficient over all participants was 0.4911, which is a moderate to strong correlation. As our data contains some outliers, i.e., panels that were viewed much longer than others (see Fig. 3), we also measured the Spearman correlation which is more robust to outliers. The results indicate a strong correlation with a median correlation coefficient of 0.7188. All correlation coefficient values were statistically significant as shown in Table 3.

One of our main findings is that the viewing time for panels is strongly positively correlated to the amount of text indicating that the individual reading speed is the most influential factor on the overall viewing time. This is generally in line with previous work on comic reading using eye tracking which found that fixations on speech bubbles are in general longer than on image regions even if they occupy smaller areas of the panels^20,21^ and that in some cases non-text panels are skipped completely^22^. However, it does not necessarily lead to the assumption that textual information is more important than the images but might rather highlight differences in processing speed of the two types of information. As initial scene processing in images is very fast^23^, it could simply be the case that readers are able to grasp the image information very efficiently while reading requires more time.

**Figure 5:**
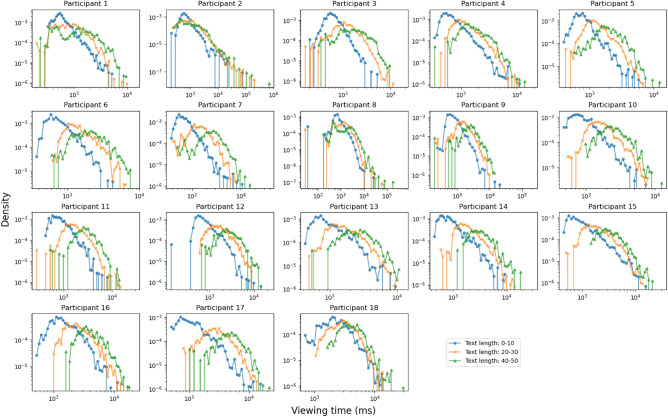
The density functions of the viewing time distributions for each participant, with speech bubble text lengths of 0 to 10, 20 to 30, and 40 to 50 characters. The tail of the distributions are approximately linear in a log-log plot, showing the characteristics of a heavy-tailed distribution.

### Image dependency

Figure 5 shows the density functions of the viewing time distributions for each participant for the short-text panels, along with panels with 20 to 30, and 40 to 50 characters are plotted as well for comparison. For short-text panels, the distribution’s tail decreases approximately linearly on a log-log plot, showing that the viewing time distribution is a heavy-tailed distribution for all participants. A similar heavy-tailed distribution emerges for panels with various text lengths as well.

The heavy-tailed distribution shows that some panels were viewed for a longer time independent of the amount of text. We assume that this may be due to some panels containing more information or showing more interesting content. Such content may either be visually interesting artwork within the comic, or a turning point in the story. Again, previous work from eye tracking points in a similar direction as the length of fixations on image regions is dependent on their content with characters being focused longer than backgrounds^20^. Additionally, it has been observed that image regions are more often fixated if panels are revisited^21^.

## Discussion

### Language dependent factors

In this paper, we analyze the viewing times of comic panels in consideration of the number of Japanese characters. Japanese characters consist of Kana and Kanji, which are phonogramic and ideogramic characters, respectively. Due to the nature of Kana and Kanji, these two types of characters carry a different amount of information, however, their processing speed is comparable^24^. Therefore in our analysis, we do not differentiate between these types of characters. Furthermore, occasional occurrences of other signs and characters including the Latin alphabet are also counted simply based on their character count. As Japanese is furthermore an agglutinative language that does not strictly define the measure of ’words’, employing metrics like the word count is also not straightforward^25^ making the character count the most suited metric for this work.

### Limitations

In the experiment, we only measure the timestamps of button presses as participants transition between comic panels. This means we cannot fully measure the attention of participants, however, we can create a natural reading experience. Specifically, we enable participants to read on their own devices at home without direct supervision and also allow them to freely choose when and how long to take breaks from reading. This way, our collected data gives a more realistic impression of how participants read comics than experiments performed in a lab setting. Furthermore, it easily enables us to collect data for complete comic volumes as participants can freely schedule their reading times which can reflect influences based on narrative processing^10^. This way, we collect viewing data for over 5,000 panels from 7 connected narratives.

One important aspect of our experiment is that the comic reading experience is altered from that of the original page-based style into a one-dimensional order, removing the two-dimensional layout information of the original panels. The panel-wise presentation removes the ability of participants to gain a quick first impression of the page content^21,26^ which could potentially influence their viewing behavior. In our interface, this may have led to more situations in which panels were re-visited compared to eye tracking results on full pages.

However, despite the loss of the two-dimensional layout, the questionnaire statistics show that the participants still found the comics understandable using our interface. As shown in Table 2, the average score of the understandability question in the questionnaire is over 3.5. We expect that this is due to the existence of an assumed reading order for each panel which is in general followed by readers^22,26^. Therefore, although the layout is altered from the original experience, we believe that our interface is able to preserve the understandability of the story.

While the panel-wise presentation does not fully capture the viewing experience of comic pages, we are able to investigate a situation closely resembling the currently emerging vertical comic readers. In these applications the screen is expected to be scrolled linearly, thus arranging the panels in a linear layout where each panel only has two direct neighbors. Therefore, our results can be used to inform insights for vertical comic readers.

As we only obtained data from 18 participants, our statistical analysis is underpowered. We were, however, able to collect a large amount of data over various comic genres per participant, which enables us to see clear tendencies per participant. Furthermore, our insights are aligned with findings from previous works indicating a stronger focus on text than images in comics^20,21^. Looking at the data between participants, we see stronger variations in individual reading speed which we assume to be the main factor for the overall viewing durations. Due to the strong variations, we assume that models to predict the viewing time of comic panels need to first assess the base reading speed of their user.

### Practical implications

Understanding comic panel viewing times has various practical applications. In particular, a model that is able to estimate a panel’s viewing time would be beneficial in various ways. One application of such a model would be an automatic comic viewer that transitions to the next panel or page without manual interaction. This idea can be further extended for the creation of a comic-to-video transformation system. Not only adding automatic transitions but also visual effects would allow to enrich the comic reading experience and could enable the automatic generation of video clips from comic books. Even when manually creating a video adaptation of the comic, the viewing time estimations may also serve as a form of assistance when assigning the amount of time to be allocated for each scene.

Based on the results, we conjecture that comic viewing times can be well described by the following simple model:

2 $$\begin{aligned} VT_{\textrm{total}}(p|r) = c(r)t(p) + i(p|r) \end{aligned}$$

where *p* is the panel, *r* is the reader of the panel, $VT_{\textrm{total}}$ is the total viewing time of a panel, *t*(*p*) is the speech bubble text length, *c*(*r*) is the reader *r*’s text reading speed, and *i*(*p*|*r*) is the time spent by the reader *r* for viewing the non-text component, i.e. the image component of the panel. The first term captures the fact that the panel viewing times show a strong linear relationship between the panel’s speech bubble text length, as shown in Fig. 3. The second term captures the image viewing time component. Since panels with fixed text lengths exhibit the same viewing time distribution as shown in Fig. 5, we expect that the second term *i*(*p*|*r*) follows the same long-tail distribution.

The coefficient *c*(*r*) can be determined for each reader *r* by linear regression, using the text length annotations available in the Manga109 dataset. It then remains to determine the function *i*(*p*|*r*), which involves specifying the relationship between the image features of the panel *p* and its viewing times,which we set as our future work.

### Future work

To gain further insights into the processing of comics and improve the proposed model, we would like to perform additional experiments and analyses. First, we would like to look more into the content of the comics and image panels. It can be expected that the reader may take time to reflect when they find interesting images or points in the story. To measure these effects, additional experiments could be performed in which participants read shorter panel sequences and annotate their opinion of the content in a panel-wise manner. Furthermore, it would be interesting to investigate in more detail why participants revisit certain panels as various factors like understanding of the plot, the quality of drawings or dialog or simply a lack of attention could contribute to this effect.

## Conclusion

In this paper, we have analyzed the statistical characteristics of the distributions of comic panel viewing times, through a user study experiment using an interface that presents comics in a panel-wise manner. Our experiments revealed that even in a bimodal setting where both images and text are concurrently present, the comic reader’s text reading speed is constant. We additionally reveal that the distribution of comic panel viewing times shares a similar heavy-tailed form between all readers. The comic panel viewing time dataset collected from our user study experiment will be released publicly for academic use. For future works, we plan to analyze the relationship between the viewing time and the panel image features, based on image processing methods.

## Data Availability

The datasets generated during and/or analysed during the current study are to be made available upon request at http://www.manga109.org/.
